# Urinary incontinence management in Chinese primary health institutions: findings from a regional survey

**DOI:** 10.7717/peerj.21079

**Published:** 2026-04-07

**Authors:** Qi Wang, Stefano Manodoro, Chaoqin Lin

**Affiliations:** 1 Department of Gynecology, Fujian Maternity and Child Health Hospital, Fuzhou, Fujian, China; 2 College of Clinical Medicine for Obstetrics & Gynecology and Pediatrics, Fujian Medical University, Fuzhou, Fujian, China; 3 Fujian Provincial Key Laboratory of Women and Children’s Critical Diseases Research, Fuzhou, Fujian, China; 4 San Paolo Hospital, ASST Santi Paolo e Carlo, Milan, Italy; 5 University of Milan, Milan, Italy

**Keywords:** Cross-sectional study, Medical training and education, Primary care system, Perceptions and clinical practices, Urinary incontinence

## Abstract

**Background:**

Urinary incontinence (UI) is a prevalent yet under-recognized condition among women in China, impairing quality of life and imposing socioeconomic burden. Primary care providers are pivotal for early detection and first-line management. Unlike Western systems dominated by general practitioners, China’ s primary women’ s health care is largely provided by gynecologists in county-level or lower-tier institutions. Evidence on their UI-related knowledge and practices is limited, hampering targeted training and policy efforts.

**Methods:**

We conducted a cross-sectional survey (March 2023–September 2024) among gynecologists in Fujian primary healthcare institutions recruited via professional WeChat groups. A structured, expert-developed questionnaire assessed demographics, UI-related awareness/perceptions (nine items, max score 18) and clinical practice (12 items, max score 24) on three-point Likert scales. Additional items explored barriers, preferred assessment methods, and factors influencing pelvic floor and bladder training. Reliability and construct validity were tested using Cronbach’s α, KMO and Bartlett’s tests. Multivariate linear regression identified factors associated with scores.

**Results:**

A total of 1,427 gynecologists responded (urban 75.5%; mean age 36.6 ± 9.3 years; 81.7% female). The questionnaire showed high reliability (α = 0.84) and good validity (KMO = 0.88; Bartlett’s *P* < 0.001). Mean awareness/perception and clinical practice scores were 12.24 ± 3.07/18 and 12.63 ± 5.41/24. Only 20.3% routinely screened for UI symptoms and 10.5% felt confident managing UI. Female gender and higher education were associated with better awareness; older age and female gender were associated with better practice (all *P* < 0.05). Lack of UI training and infrequent literature reading correlated with lower scores. Urban physicians cited time constraints, whereas rural physicians cited limited space, lack of feedback mechanisms for exercises, and lower use of pelvic floor ultrasound or pad tests.

**Conclusions:**

This first large-scale evaluation of UI-related knowledge and practice among Chinese primary care gynecologists reveals substantial gaps, especially in rural areas. Incorporating UI into continuing medical education, promoting routine screening, updating diagnostic knowledge, and applying digital tools for real-time monitoring of pelvic floor training may improve early detection and care. Tailored interventions addressing urban–rural disparities are essential to strengthen UI management at the primary care level.

## Introduction

Urinary incontinence (UI) is a common medical condition in women. Globally, contemporary epidemiological studies indicate that approximately 25–45% of adult women experience some degree of UI, with prevalence increasing with age, parity, obesity, and menopausal status, making UI one of the most prevalent chronic conditions affecting women worldwide (Milsom & Gyhagen, 2019; Irwin et al., 2011; Abrams et al., 2018). Recently, a large-scale epidemiological survey was carried out in southeastern China, which revealed that nearly one-fourth (24.8%) of women had UI, and the most common subtype was stress urinary incontinence (SUI) (Wang et al., 2023a; Wang et al., 2023b). UI not only impairs the quality of life but also induces anxiety, depression, and social isolation, leading to a heavy socioeconomic burden through increased healthcare utilization, productivity loss, and long-term care costs (Riss & Kargl, 2011; Felde, Bjelland & Hunskaar, 2012; Hansson Vikström et al., 2021; Yip et al., 2013; Wang et al., 2023a; Wang et al., 2023b). Large national surveys further indicate that approximately 16% of adult Chinese women experience UI, corresponding to over 85 million affected individuals; however, help-seeking behavior remains low and substantial unmet clinical needs persist (Zhu et al., 2024).

Given the growing burden of UI, primary healthcare providers are increasingly recognized as essential for early detection. In developed countries, researchers advocate for routine screening by primary care physicians (PCPs) to improve disclosure rates, reduce treatment delays, and facilitate access to specialized care (O’Reilly et al., 2018; Luebke et al., 2024). Moreover, PCPs play a crucial role in guiding and supervising first-line UI treatments, including lifestyle modifications, pelvic floor muscle training, and behavioral therapy, as recommended by several international guidelines (National Institute for Health and Care Excellence (NICE), 2019; Lightner et al., 2019; Nambiar et al., 2018). Despite guideline recommendations and the high prevalence of UI, UI remains substantially under-recognized and undertreated in many primary care systems worldwide, with low screening and diagnosis rates and documented gaps in provider knowledge and practice (Geynisman-Tan et al., 2024; Patel et al., 2023).

Unlike in many Western healthcare systems where primary care is delivered predominantly by general practitioners (GPs) with doctoral-level training, China’s primary care for women’s health is often provided by gynecologists working in county-level or lower-tier institutions. These clinicians are specialists in gynecology, but they function as front-line providers for female-specific conditions such as UI. China’s primary healthcare system is characterized by notable heterogeneity in workforce distribution, institutional resources, and service capacity, with rural areas generally having fewer trained personnel, lower access to continuing education, and less infrastructure compared with urban regions (Wang et al., 2020). Due to disparities in medical education and healthcare infrastructure—especially in rural areas—many of these physicians have associate degrees or vocational training, in contrast to their urban counterparts who are more likely to hold bachelor’s or master’s degrees.

However, current evidence regarding the perceptions, clinical practices, and management capabilities of Chinese primary care gynecologists in UI care remains limited. Most existing research in this field has focused either on population-level epidemiology or on women’s experiences of UI, while systematic evaluation of provider-level knowledge, attitudes, and clinical practice within China’s primary care system remains scarce (Pang et al., 2025; Zhu et al., 2024). This knowledge gap hinders the development of targeted training programs and healthcare policies aimed at improving UI outcomes at the primary care level. Insights from China’s experience, where gynecologists serve dual roles in primary and specialty care, may provide transferable lessons for other health systems facing shortages of GPs or primary care infrastructure.

Therefore, this study was designed to address the following research question: What are the current levels of awareness, perceptions, and clinical practices related to UI among primary care gynecologists in China, and what individual, institutional, and systemic factors, including resource-related barriers, influence the quality and accessibility of UI management across urban and rural primary care settings?

To this end, we conducted a large-scale survey in Fujian Province to evaluate UI-related awareness, perceptions, and clinical practices among primary care providers and to identify associated factors and key barriers to effective management in both urban and rural settings. These findings are expected to inform future capacity-building initiatives and health policy development tailored to the realities of primary care in low-resource settings.

## Methods

### Study design and participants

This large-scale cross-sectional survey was conducted between March 2023 and September 2024 within a provincial maternal and child health professional network in Fujian Province, which served as the organizational framework for collaboration among primary healthcare institutions.

Eligible institutions were county-level or lower-tier primary healthcare facilities that routinely provided gynecological services and were registered within this professional network. Institutions not meeting these criteria were not included. Eligible participants were licensed gynecologists actively practicing in these participating county-level or lower-tier institutions at the time of the survey; physicians not engaged in routine clinical care were excluded.

To maximize geographic representativeness, all eligible institutions within the network across Fujian Province were invited to participate, thereby ensuring inclusion of both urban and rural primary care settings. However, because recruitment relied on a convenience sampling strategy with voluntary participation, the urban-rural distribution of respondents was not pre-specified or controlled.

Participants were recruited *via* electronic invitations distributed through professional WeChat groups coordinated by the participating institutions. Although this approach does not constitute probability sampling, it enabled efficient large-scale recruitment and broad geographic coverage. Potential selection bias associated with voluntary participation is acknowledged in the limitations section.

This study received ethical approval from the Ethics Committee of Fujian Maternity and Child Health Hospital (Approval No. 2024KY268). Written informed consent was obtained from all participants prior to data collection. All participant data were de-identified using numerical coding systems in accordance with applicable data protection regulations and the principles of the Declaration of Helsinki. The anonymized data were stored on password-protected computers with access restricted to authorized research personnel only.

### Data collection

The questionnaire was originally developed in Chinese based on the consensus of the Provincial Urogynecology Academic Committee, utilizing a two-round Delphi consultation process. Prior to the formal survey, a pilot test was conducted among 50 primary care gynecologists to evaluate the clarity and readability, and minor revisions were made accordingly. The original questionnaire was in Chinese and the corresponding English version is provided in Appendix 1.

The questionnaire consisted of the following sections: ① Demographics and professional background (age, gender, years of practice, location of workplace, education level, prior training in UI management, and literature reading experience); ② Awareness and perceptions assessment (nine items) evaluating UI social perceptions, and attitudes toward patient care; ③ Clinical practice assessment (12 items) covering UI assessment and management strategies.

Responses in sections ② and ③ were rated on a three-point Likert scale. The awareness and perceptions section had a maximum score of 18, the clinical practice section had a maximum score of 24. Higher scores reflect greater awareness of UI, more favorable attitudes toward UI management, and stronger adherence to recommended clinical practices.

Additionally, three multiple-choice questions were included to explore barriers to specialized UI examinations, preferred assessment methods, and factors influencing efficacy of pelvic floor muscle training and bladder training.

### Statistical analysis

In this study, SPSS software and the relevant package of R software were applied to analyze the data statistically. Continuous data was presented as mean ± standard deviation (SD) and between-group comparison was made using Student’s *t*-test. Categorical data was presented as counts (percentages) (n (%)) and was compared using Pearson’s *χ*^2^ test. Variables with a *P*-value < 0.05 in univariate analysis and those considered theoretically relevant were entered into the multivariate linear regression model. Multiple linear regression analysis was employed to establish independently associated variables with awareness and perceptions scores, clinical practice scores, and overall scores while adjusting for confounders. Results were presented as regression coefficients (β) along with accompanying 95% confidence intervals (CIs). Statistical tests were all two-tailed with a statistically significant threshold set at *P* < 0.05.

Reliability of the questionnaire was assessed using Cronbach’s α to measure internal consistency. A Cronbach’s α of ≥ 0.70 was considered adequate and indicated good reliability. Construct validity of the questionnaire was assessed by factor analysis. Kaiser-Meyer-Olkin (KMO) test was employed to test sampling adequacy, and a KMO ≥ 0.70 was considered suitable for factor analysis. Bartlett’s test of sphericity was performed to check whether the correlation matrix was applicable in factor analysis. A significant Bartlett’s test, with *P* < 0.05, assured that the data were suitable for further multivariate analysis.

## Results

### Demographic and professional characteristics of participants

A total of 1,427 primary care gynecologists participated, with 75.5% practicing in urban areas. The mean age was 36.61 ± 9.29 years, and 81.7% were female, with a significantly higher proportion of women in urban areas (*P* = 0.004). Most participants held a bachelor’s (54.7%) or associate degree (35.1%), and urban physicians had higher educational level (*P* < 0.001). Approximately 44.4% had received UI-specific training in the past five years, with urban physicians more likely to have undergone training (*P* = 0.037). Further details are in Table 1.

**Table 1:** Demographic professional background characteristics of participants.

Characteristics	Overall (*n* = 1,427)	Urban (*n* = 1,078)	Rural (*n* = 349)	t/*χ*^2^	*P* values
Age, yrs, (mean ± SD)	36.61 ± 9.29	36.87 ± 9.32	35.80 ± 9.17	1.864	0.063
Gender, n (%)				8.377	0.004
Male	261 (18.3)	179 (16.6)	82 (23.5)		
Female	1,166 (81.7)	899 (83.4)	267 (76.5)		
Educational level, n (%)				43.351	<0.001
Technical secondary school	108 (7.6)	58 (5.4)	50 (14.3)		
Associate degree	501 (35.1)	365 (33.9)	136 (39.0)		
Bachelor’s degree	781 (54.7)	620 (57.5)	161 (46.1)		
Master’s degree	37 (2.6)	35 (3.2)	2 (0.6)		
Clinical experience duration, yrs, n (%)				5.265	0.261
<5	258 (18.1)	185 (17.2)	73 (20.9)		
5–10	318 (22.3)	238 (22.1)	80 (22.9)		
10–20	496 (34.8)	384 (35.6)	112 (32.1)		
20–30	239 (16.7)	177 (16.4)	62 (17.8)		
>30	116 (8.1)	94 (8.7)	22 (6.3)		
UI-specific training (past 5 yrs), n (%)				4.344	0.037
Yes	633 (44.4)	495 (45.9)	138 (39.5)		
No	794 (55.6)	583 (54.1)	211 (60.5)		
UI literature studied (past 5 yrs), n (%)				0.022	0.883
Yes	843 (59.1)	638 (59.2)	205 (58.7)		
No	584 (40.9)	440 (40.8)	144 (41.3)		

**Notes.**

Abbreviations SD standard deviation UI urinary incontinence

### Awareness and perceptions of UI

The mean awareness and perception score was 12.24 ± 3.07 (out of 18). Despite no significant difference urban-rural in total awareness scores (*P* = 0.263), rural physicians demonstrated specific misconceptions and avoidance behaviors that suggest targeted educational interventions are needed. Rural physicians were more likely to underestimate UI prevalence and its impact on daily life (*P* = 0.008 and *P* = 0.012, respectively). Rural physicians were significantly more likely to avoid UI discussions than urban physicians (7.2% *vs.* 4.3%, *P* = 0.041). Although only 10.2% perceived UI as a natural part of aging, 36.3% partially agreed. Additionally, 75.3% believed UI should be actively managed. See Table 2 for full results on awareness, perceptions, and clinical practice differences.

**Table 2:** Detailed evaluation of UI perception and clinical practice.

Characteristics, n (%)	Overall (*n* = 1,427)	Urban (*n* = 1078)	Rural (*n* = 349)	t/*χ*^2^	*P* values
**Awareness and Perceptions**					
1. Do you perceive UI as an uncommon condition among women?				9.609	0.008
Agree	314 (22.0)	240 (22.3)	74 (21.2)		
Partially agree	699 (49.0)	505 (46.8)	194 (55.6)		
Disagree	414 (29.0)	333 (30.9)	81 (23.2)		
2. Do you think that discussing UI with patients makes them feel embarrassed?				0.405	0.817
Agree	212 (14.9)	160 (14.8)	52 (14.9)		
Partially agree	678 (47.5)	517 (48)	161 (46.1)		
Disagree	537 (37.6)	401 (37.2)	136 (39.0)		
3. Do you avoid discussing topics related to UI with patients?				6.388	0.041
Agree	71 (5.0)	46 (4.3)	25 (7.2)		
Partially agree	404 (28.3)	298 (27.6)	106 (30.4)		
Disagree	952 (66.7)	734 (68.1)	218 (62.5)		
4. Do you believe that if a patient does not proactively mention UI, it may not significantly affect her?				0.233	0.890
Agree	108 (7.6)	83 (7.7)	25 (7.2)		
Partially agree	577 (40.4)	438 (40.6)	139 (39.8)		
Disagree	742 (52.0)	557 (51.7)	185 (53)		
5. Do you think that UI does not significantly impact patients’ daily lives and social activities?				8.796	0.012
Agree	50 (3.5)	39 (3.6)	11 (3.2)		
Partially agree	330 (23.1)	229 (21.2)	101 (28.9)		
Disagree	1,047 (73.4)	810 (75.2)	237 (67.9)		
6. Do you believe that UI does not significantly affect patients’ sexual lives?				2.435	0.296
Agree	50 (3.5)	37 (3.4)	13 (3.7)		
Partially agree	330 (23.1)	239 (22.2)	91 (26.1)		
Disagree	1,047 (73.4)	802 (74.4)	245 (70.2)		
7. Do you perceive UI as a natural part of aging rather than a medical condition?				0.675	0.714
Agree	146 (10.2)	112 (10.4)	34 (9.7)		
Partially agree	518 (36.3)	385 (35.7)	133 (38.1)		
Disagree	763 (53.5)	581 (53.9)	182 (52.1)		
8. Do you think that little can be done about UI, so there is no need to spend time managing it?				1.779	0.411
Agree	42 (2.9)	32 (3.0)	10 (2.9)		
Partially agree	311 (21.8)	226 (21.0)	85 (24.4)		
Disagree	1,074 (75.3)	820 (76.1)	254 (72.8)		
9. Do you think that patients should be guided to understand that UI is a natural phenomenon of aging and learn to adapt to it?				1.228	0.541
Agree	181 (12.7)	140 (13.0)	41 (11.7)		
Partially agree	502 (35.2)	371 (34.4)	131 (37.5)		
Disagree	744 (52.1)	567 (52.6)	177 (50.7)		
**Awareness and Perceptions Score**, (mean ± SD)	12.24 ± 3.07	12.29 ± 3.02	12.08 ± 3.20	1.119	0.263
**Clinical Practice**					
10. Do you confirm the presence of UI symptoms when consulting patients?				6.050	0.049
Rarely	583 (40.9)	449 (41.7)	134 (38.4)		
Sometimes	553 (38.8)	399 (37.0)	154 (44.1)		
Often	291 (20.3)	230 (21.3)	61 (17.5)		
11. When patients mention UI, do you inquire about its impact on their quality of life?				4.078	0.130
Rarely	190 (13.3)	144 (13.4)	46 (13.2)		
Sometimes	591 (41.4)	431 (40.0)	160 (45.8)		
Often	646 (45.3)	503 (46.7)	143 (41.0)		
12. Do you provide questionnaires such as ICIQ-SF, I-QOL, or PISQ-12 to assess the impact of UI on various aspects of quality of life?				2.047	0.359
Rarely	192 (13.5)	150 (13.9)	42 (12.0)		
Sometimes	650 (45.5)	480 (44.5)	170 (48.7)		
Often	585 (41.0)	448 (41.6)	137 (39.3)		
13. Are you confident in managing UI effectively?				1.091	0.580
Agree	149 (10.5)	112 (10.4)	37 (10.6)		
Partially agree	854 (59.8)	638 (59.2)	216 (61.9)		
Disagree	424 (29.7)	328 (30.4)	96 (27.5)		
14. Do you have a clear understanding of the causes of UI?				4.082	0.130
Agree	343 (24.0)	273 (25.3)	70 (20.1)		
Partially agree	876 (61.4)	649 (60.2)	227 (65.0)		
Disagree	208 (14.6)	156 (14.5)	52 (14.9)		
15. Can you differentiate the type and severity of UI in diagnosis?				7.195	0.027
Agree	287 (20.1)	228 (21.2)	59 (16.9)		
Partially agree	849 (59.5)	620 (57.5)	229 (65.6)		
Disagree	291 (20.4)	230 (21.3)	61 (17.5)		
16. Can you develop a diagnostic and treatment plan for managing patients with UI?				0.617	0.734
Agree	265 (18.6)	196 (18.2)	69 (19.8)		
Partially agree	788 (55.2)	601 (55.8)	187 (53.6)		
Disagree	374 (26.2)	281 (26.1)	93 (26.6)		
17. Do you perform specialized physical examinations (*e.g.*, stress test) for patients with UI?				12.955	0.002
Rarely	586 (41.0)	434 (40.3)	152 (43.6)		
Sometimes	563 (39.5)	411 (38.1)	152 (43.6)		
Often	278 (19.5)	233 (21.6)	45 (12.9)		
18. Do you understand how to conduct a pad test and explain it to patients?				0.243	0.886
Agree	347 (24.3)	264 (24.5)	83 (23.8)		
Partially agree	777 (54.5)	583 (54.1)	194 (55.6)		
Disagree	303 (21.2)	231 (21.4)	72 (20.6)		
19. Do you understand how to conduct a voiding diary and explain it to patients?				2.320	0.313
Agree	381 (26.7)	292 (27.1)	89 (25.5)		
Partially agree	749 (52,5)	554 (51.4)	195 (55.9)		
Disagree	297 (20.8)	232 (21.5)	65 (18.6)		
20. Do you understand how to perform Kegel exercises and explain them to patients?				1.257	0.533
Agree	522 (36.6)	403 (37.4)	119 (34.1)		
Partially agree	704 (49.3)	526 (48.8)	178 (51.0)		
Disagree	201 (14.1)	149 (13.8)	52 (14.9)		
21. Do you have confidence in the effectiveness of health education for UI patients, such as teaching Kegel exercises and bladder training to improve symptoms?				0.080	0.961
Agree	600 (42.0)	451 (41.8)	149 (42.7)		
Partially agree	682 (47.8)	517 (48.0)	165 (47.3)		
Disagree	145 (10.2)	110 (10.2)	35 (10.0)		
**Clinical Practice Score**, (mean ± SD)	12.63 ± 5.41	12.69 ± 5.53	12.46 ± 5.03	0.706	0.480
**Total Score**, (mean ± SD)	24.87 ± 6.04	24.97 ± 6.17	24.54 ± 5.62	1.227	0.220

**Notes.**

Abbreviations UI urinary incontinence SD standard deviation ICIQ-SF International Consultation on Incontinence Questionnaire Urinary Incontinence Short Form I-QOL Incontinence Quality of Life Questionnaire PISQ-12 Pelvic Organ Prolapse/Urinary Incontinence Sexual Function Questionnaire (Short Form)

### Clinical practice in UI management

The mean clinical practice score was 12.63 ± 5.41 (out of 24), with poor overall performance in UI screening, diagnosis, and management. Only 20.3% of physicians routinely inquired about UI symptoms, and rural physicians were less likely to actively screen for UI (*P* = 0.049). Confidence in UI management was low, with only 10.5% of respondents feeling capable of managing UI effectively. Rural physicians expressed less confidence in differentiating UI types and assessing severity (*P* = 0.027). They also much less frequently performed specialized physical examinations than urban physicians (*P* = 0.002).

### Questionnaire reliability and validity

Internal consistency of the questionnaire was evaluated using the full dataset of 1,427 respondents. Overall Cronbach’s α indicated high reliability (overall Cronbach’s α = 0.84). The awareness and perceptions domain had a Cronbach’s α of 0.73, which indicates fair internal consistency, and clinical practice domain reflected high internal consistency (Cronbach’s α = 0.89).

Construct validity was confirmed by factor analysis. The KMO measure was 0.88, indicating very good sampling adequacy. Bartlett’s test of sphericity was significant (*χ*^2^ = 12,652.1, *df* = 210, *P* < 0.001), confirming that data were sufficient for further multivariate analysis.

### Factors associated with awareness, perceptions and clinical practice scores

Regression analysis revealed that higher awareness scores were significantly associated with female gender (*P* = 0.038), bachelor’s (*P* = 0.007) and master’s degrees (*P* = 0.006). Higher clinical practice scores correlated positively with age (*P* = 0.011) and female gender (*P* < 0.001), while lack of UI training (*P* < 0.001) and absence of UI literature reading (*P* < 0.001) were linked to lower scores. Total scores followed a similar trend (Table 3).

**Table 3:** Outcomes of multi-variable regression.

Characteristics	β	95% CI	*P* values
**Awareness and Perceptions Score**			
Age	0.03	(−0.02, 0.07)	0.220
Gender			
Male	Ref.		
Female	0.46	(0.03, 0.89)	0.038
Educational level			
Technical secondary school	Ref.		
Associate degree	0.34	(−0.3, 0.98)	0.298
Bachelor’s degree	0.86	(0.24, 1.49)	0.007
Master’s degree	1.62	(0.47, 2.76)	0.006
Clinical experience duration			
<5	Ref.		
5–10	−0.29	(−0.83, 0.24)	0.282
10–20	−0.52	(−1.18, 0.15)	0.129
20–30	0.40	(−0.58, 1.37)	0.424
>30	−0.81	(−2.14, 0.51)	0.228
Region			
Urban	Ref.		
Rural	−0.05	(−0.42, 0.32)	0.799
UI-specific training (past 5 yrs)			
Yes	Ref.		
No	−0.31	(−0.69, 0.06)	0.101
UI literature studied (past 5 yrs)			
Yes	Ref.		
No	−0.31	(−0.69, 0.07)	0.110
**Clinical Practice Score**			
Age	0.08	(0.02, 0.15)	0.011
Gender			
Male	Ref.		
Female	2.49	(1.8, 3.18)	<0.001
Educational level			
Technical secondary school	Ref.		
Associate degree	−0.63	(−1.65, 0.4)	0.232
Bachelor’s degree	−0.46	(−1.46, 0.54)	0.371
Master’s degree	−0.70	(−2.54, 1.13)	0.454
Clinical experience duration			
<5	Ref.		
5–10	0.24	(−0.62, 1.1)	0.583
10–20	−0.21	(−1.28, 0.85)	0.695
20–30	−0.26	(−1.81, 1.29)	0.743
>30	−0.28	(−2.4, 1.84)	0.794
Region			
Urban	Ref.		
Rural	0.13	(−0.46, 0.72)	0.671
UI-specific training (past 5 yrs)			
Yes	Ref.		
No	−2.08	(−2.68, −1.48)	<0.001
UI literature studied (past 5 yrs)			
Yes	Ref.		
No	−2.46	(−3.07, −1.85)	<0.001
**Total Score**			
Age	0.11	(0.04, 0.18)	0.003
Gender			
Male	Ref.		
Female	2.95	(2.19, 3.71)	<0.001
Educational level			
Technical secondary school	Ref.		
Associate degree	−0.29	(−1.42, 0.84)	0.620
Bachelor’s degree	0.41	(−0.7, 1.51)	0.470
Master’s degree	0.92	(−1.1, 2.93)	0.374
Clinical experience duration			
<5	Ref.		
5–10	−0.05	(−1, 0.89)	0.910
10–20	−0.73	(−1.9, 0.44)	0.222
20–30	0.14	(−1.57, 1.84)	0.876
>30	−1.10	(−3.43, 1.23)	0.356
Region			
Urban	Ref.		
Rural	0.08	(−0.57, 0.73)	0.809
UI-specific training (past 5 yrs)			
Yes	Ref.		
No	−2.40	(−3.06, −1.74)	<0.001
UI literature studied (past 5 yrs)			
Yes	Ref.		
No	−2.77	(−3.44, −2.1)	<0.001

**Notes.**

Abbreviations CI confidence interval Ref reference UI urinary incontinence

### Barriers and preferences in UI management

The primary barrier to conducting specialized UI examinations was lack of proficiency (71.5%), with no urban–rural difference. Limited clinic space was a more significant issue in rural areas (*P* = 0.008), whereas time constraints were reported more frequently in urban settings (*P* = 0.031). Preferred assessment methods included pelvic floor ultrasound (75.8%) and the one-hour pad test (70.2%), though utilization rates were lower in rural areas (*P* = 0.006). The most frequently reported challenge in conservative management was the inability to monitor patient adherence (78.0%), more prominent in rural areas (*P* = 0.040). Additionally, urban physicians more frequently cited insufficient consultation time as a barrier to properly instructing patients on these exercises (*P* = 0.016), while rural physicians were more concerned about the lack of tools to monitor treatment efficacy and adjust strategies accordingly (*P* = 0.035). Detailed results are in Table 4.

**Table 4:** Summary of multiple-choice survey results.

Questions & Options	Overall	Urban	Rural	*χ* ^2^	*P* values
1. Barriers to performing specialized physical examinations or tests	*n* = 1,149	*n* = 845	*n* = 304		
Lack of proficiency in performing the procedure	821 (71.5)	604 (71.5)	217 (71.4)	0.001	0.974
Perceived lack of necessity	259 (22.5)	194 (23.0)	65 (21.4)	0.318	0.573
Limited clinic space	594 (51.7)	417 (49.3)	177 (58.2)	7.051	0.008
Time constraints during consultations	468 (40.7)	360 (42.6)	108 (35.5)	4.639	0.031
Mobility limitations in elderly patients	406 (35.3)	294 (34.8)	112 (36.8)	0.411	0.522
Other reasons	147 (12.8)	115 (13.6)	32 (10.5)	1.905	0.168
2. Preferred assessments for urinary incontinence	*n* = 265	*n* = 196	*n* = 69		
1-hour pad test	186 (70.2)	142 (72.4)	44 (63.8)	1.838	0.175
Urodynamic test	184 (69.4)	145 (74.0)	39 (56.5)	7.329	0.007
Urinalysis	160 (60.4)	122 (62.2)	38 (55.1)	1.097	0.295
Blood glucose test	69 (26.0)	50 (25.5)	19 (27.5)	0.109	0.742
Urinary tract ultrasound	162 (61.1)	122 (62.2)	40 (58.0)	0.392	0.531
Pelvic floor ultrasound	201 (75.8)	157 (80.1)	44 (63.8)	7.433	0.006
Voiding diary	157 (59.2)	116 (59.2)	41 (59.4)	0.001	0.973
Other assessments	14 (5.3)	8 (4.1)	6 (8.7)	2.262	0.133
3. Factors affecting the efficacy of Kegel exercises and bladder training	*n* = 1,427	*n* = 1,078	*n* = 349		
Insufficient consultation time to properly instruct patients	835 (58.5)	650 (60.3)	185 (53.0)	5.769	0.016
Explanations are difficult for patients to understand	917 (64.3)	703 (65.2)	214 (61.3)	1.742	0.187
Limited space and conditions for proper guidance	823 (57.7)	619 (57.4)	204 (58.5)	0.115	0.735
Lack of effective methods to supervise patients’ adherence	1,113 (78.0)	827 (76.7)	286 (81.9)	4.206	0.040
Lack of tools to monitor treatment efficacy and adjust strategies accordingly	952 (66.7)	703 (65.2)	249 (71.3)	4.466	0.035
Other factors	118 (8.3)	94 (8.7)	24 (6.9)	1.181	0.377

**Notes.**

Notes: Data are presented as frequency (percentage).

## Discussion

This study systematically evaluated the awareness, perceptions, and clinical practices of primary care gynecologists in Fujian Province regarding UI management, highlighting persistent misconceptions, suboptimal clinical confidence, and disparities between urban and rural physicians. Although most physicians recognized UI as a medical condition, nearly half held misconceptions about its inevitability with aging, and a significant proportion underestimated its impact on quality of life. Rural physicians were more likely to avoid discussing UI with patients, reflecting potential gaps in patient education and counseling.

Clinical practice performance was generally inadequate, with low rates of routine UI screening and limited confidence in UI diagnosis and management. Only 20.3% of physicians routinely inquired about UI symptoms, and fewer than one-fifth felt capable of developing a comprehensive UI treatment plan. Rural physicians demonstrated lower diagnostic confidence and were less likely to perform specialized examinations, likely due to training deficits and resource constraints.

Our multivariate analysis identified key factors influencing UI perception and practice, including gender, education level, and training exposure. Female physicians and those with higher education levels exhibited better awareness and clinical competency, whereas lack of UI training and limited literature exposure were strongly associated with lower scores. Barriers such as inadequate procedural proficiency, time constraints, and limited access to training further hindered effective UI management, particularly in rural settings.

To our knowledge, no previous studies have directly assessed UI awareness, perceptions and clinical practice among primary care gynecologists in China. In contrast, due to differences in healthcare system structures, the role of Chinese primary care gynecologists in UI management is often partially fulfilled by primary PCPs in many other countries. As a result, existing literature on UI in primary care has primarily focused on PCPs. Given their accessibility, PCPs are often the first point of contact for women seeking UI care (Shaw et al., 2006). Furthermore, because non-surgical UI management requires long-term follow-up and physician-patient interaction, PCPs play a key role in early screening, conservative treatment, and patient education (Kirby, 2006). As early as Jolleys (1989) reported that general practitioners (GPs) could diagnose and improve symptoms in most women with UI through appropriate interventions. Similarly, in 1996, Sandvik & Hunskaar (1995) documented that in Bergen, Norway, 12% of women with UI underwent a stress test, and 73% received a urinalysis from their GPs. More recently, a 2020 survey of Nordic GPs highlighted that the greatest barrier to UI management was the “uncertainty of how to treat UI”. The study also found that female physicians were more likely to engage in UI discussions with patients, consistent with our findings (Elsner et al., 2020). Likewise, a 2024 U.S. study reported that PCPs had a low UI screening rate, with only 42% screening at least half of their female patients. None of the surveyed physicians felt fully confident in their UI screening ability, and 16% of annual health check records lacked any documentation of UI screening (Geynisman-Tan et al., 2024). These findings align with our study, suggesting that UI remains an underrecognized and undertreated condition even within primary healthcare settings.

In addition to evidence from primary care settings, studies from other healthcare and community contexts further support the widespread lack of awareness and knowledge regarding UI. A scoping review by Van Vuuren et al. (2021) demonstrated considerable variability and frequent deficiencies in healthcare practitioners’ knowledge, attitudes, and practices toward UI management across diverse clinical settings. From the patient and public perspective, Roe, Wilson & Doll (2001) and Saleh et al. (2005) reported that insufficient information, social stigma, and limited access to structured continence services substantially hindered appropriate care-seeking and effective management of UI. Moreover, the development of standardized tools such as the Urinary Incontinence Awareness and Attitude Scale (URINAS) (Avci, Yıldırım & Eren, 2022) reflects growing recognition of the need to systematically assess awareness and attitudes toward UI. Collectively, these findings provide important contextual support for our results and further emphasize the significance of addressing UI-related knowledge and practice gaps at the level of primary care providers.

Augmenting UI management by primary care gynecologists requires a multi-faceted approach addressing education, realignment of resources, and digital health integration. The lack of significant participation of primary care gynecologists in training programs addressing UI necessitates UI management’s inclusion in continuing medical education (CME) curriculum. Moreover, the inclusion of UI screening in routine gynecological check-ups and encouraging physicians to proactively inquire about UI symptoms would increase detection rates at an early stage. Use of standardized instruments, such as the ICIQ-SF, should also be promoted to provide uniform assessments.

Our findings suggest that the high preference for urodynamic testing among primary care gynecologists may reflect outdated knowledge rather than evidence-based practice. Current international guidelines consensus emphasize that urodynamic testing should be reserved for complicated UI, rather than as routine diagnostic tools (Ruffolo et al., 2024; Nambiar et al., 2017). This finding indicated efforts should focus on updating physician knowledge to align with evidence-based UI assessment and management strategies.

In addition, many physicians in our study reported that it was difficult to track patient adherence to exercises and evaluate efficacy, with urban physicians reporting time shortage in patient education. The application of digital health technologies such as smartphone-based applications and wearables can reinforce UI management with real-time tracking of symptoms, voiding diary tracking, pelvic floor muscle exercise, and custom feedback (Ben Arous et al., 2023). These technologies can support patient compliance and optimize treatment effect, particularly among resource-limited rural communities.

This study has several limitations that should be acknowledged. First, the cross-sectional design does not allow causal inference; therefore, the associations observed between provider characteristics, institutional factors, and UI management practices should be interpreted as correlational. Second, the use of self-reported survey data may be subject to response bias, as physicians who are more knowledgeable or confident in UI management may have been more likely to participate, potentially leading to an overestimation of overall competence. Third, because participation was voluntary and anonymous and no comprehensive registry of primary care gynecologists was available, an accurate response rate could not be calculated, limiting precise assessment of sample representativeness. Although the study achieved broad geographic coverage across Fujian Province and included both urban and rural primary care institutions, generalisability to other regions of China or to healthcare systems with different organisational structures may therefore be constrained. Finally, while the questionnaire was developed based on expert consensus and existing literature, it has not undergone formal psychometric validation. Future research should aim to develop and validate standardized assessment instruments specifically tailored to the Chinese primary care context to further strengthen measurement reliability.

## Conclusions

This study provides the first large-scale assessment of UI-related awareness, perceptions, and clinical practice among primary care gynecologists in China, revealing substantial gaps in knowledge, confidence, and routine management, particularly in rural settings. These findings underscore the urgent need for targeted system-level interventions, including strengthened continuing medical education, integration of routine UI screening into primary care, and improved access to training and diagnostic resources. Addressing structural barriers such as limited clinical space in rural institutions and time constraints in urban practices may further enhance the effectiveness of UI management. Collectively, these measures have the potential to significantly improve early detection, treatment quality, and health outcomes for women with UI in China and in comparable healthcare systems.

##  Supplemental Information

10.7717/peerj.21079/supp-1 Supplemental Information 1 Full dataset containing all collected responses

10.7717/peerj.21079/supp-2 Supplemental Information 2 Study Questionnaire (Chinese and English Versions)

10.7717/peerj.21079/supp-3 Supplemental Information 3 STROBE checklist

## Additional Information and Declarations

### Data Availability

The following information was supplied regarding data availability: The raw data is available in the Supplemental File.
