# Supplementary material for: Urinary incontinence management in Chinese primary health institutions: findings from a regional survey
Source: PeerJ. 2026 Apr 7;14:e21079. doi: 10.7717/peerj.21079 (PMC13068013; doi:10.7717/peerj.21079)
Supplement: Supplemental Information 2 [file peerj-14-21079-s002.docx]

**Appendix 1: Study Questionnaire (Chinese and English Versions)**

This structured self-administered questionnaire was developed by the Urogynecology Expert Panel of the Fujian Smart Pelvic Floor Health Alliance. The questionnaire consists of three sections: demographics and professional background, awareness and perceptions assessment, and clinical practice assessment. Additionally, three multiple-choice questions were included to explore barriers to specialized urinary incontinence (UI) examinations, preferred assessment methods, and factors influencing efficacy to pelvic floor muscle training and bladder training.

本问卷由福建省盆底健康智能联盟的女性泌尿妇科医学专家团队制定，包括三部分内容：人口统计信息及职业背景、对尿失禁（UI）的认知和看法评估、以及临床实践评估。此外，问卷包含三个多项选择题，以探讨专业尿失禁检查的障碍、倾向的评估方法以及影响盆底肌训练和膀胱训练效果的因素。

**Section 1: Demographics and Professional Background**

**第1部分：人口统计信息及职业背景**

1. What is your age? / 你的年龄是？
2. What is your gender? / 你的性别是？
   ① Male / 男
   ② Female / 女
3. What is your highest level of education? / 你的最高学历是？
   ① Technical Secondary School / 中专
   ② Associate / 大专
   ③ Bachelor / 本科
   ④ Master / 硕士
   ⑤ Doctor / 博士
4. How many years have you been practicing? / 你的执业年限是？
   ① <5 years / <5 年
   ② 5-10 years / 5-10 年
   ③ 10-20 years / 10-20 年
   ④ 20-30 years / 20-30 年
   ⑤ >30 years / >30 年
5. Could you please indicate whether your medical practice is located in an urban or rural area? / 你的执业医疗机构位于城区还是乡镇？
   ① Urban / 城市
   ② Rural / 农村
6. Have you attended any specialized training programs on female UI in the past five years? / 过去五年内，你是否接受过女性尿失禁的专业培训？
   ① Yes / 是
   ② No / 否
7. Have you read any professional literature on female UI in the past five years? / 过去五年内，你是否阅读过女性尿失禁的专业文献？
   ① Yes / 是
   ② No / 否

**Section 2: Awareness and Perceptions of UI (Scored on a 0-18 scale)**

**第2部分：对尿失禁的认知和看法（总分0-18）**

Q1. Do you perceive UI as an uncommon condition among women?
您认为尿失禁在女性中是不常见的情况吗？
① Agree / 同意
② Partially agree / 部分同意
③ Disagree / 不同意

Q2. Do you think discussing UI with patients makes them feel embarrassed?
您认为与患者讨论尿失禁会让她们感到尴尬吗？
① Agree / 同意
② Partially agree / 部分同意
③ Disagree / 不同意

Q3. Do you avoid discussing topics related to UI with patients?
您是否会避免与患者讨论尿失禁相关话题？
① Agree / 同意
② Partially agree / 部分同意
③ Disagree / 不同意

Q4. Do you believe that if a patient does not proactively mention UI, it may not significantly affect her?
您是否认为如果患者没有主动提及尿失禁，说明该问题对她影响不大？
① Agree / 同意
② Partially agree / 部分同意
③ Disagree / 不同意

Q5. Do you think UI does not significantly impact patients’ daily lives and social activities?
您认为尿失禁对患者的日常生活和社交活动影响不大吗？
① Agree / 同意
② Partially agree / 部分同意
③ Disagree / 不同意

Q6. Do you believe UI does not significantly affect patients’ sexual lives?
您认为尿失禁对患者的性生活影响不大吗？
① Agree / 同意
② Partially agree / 部分同意
③ Disagree / 不同意

Q7. Do you perceive UI as a natural part of aging rather than a medical condition?
您认为尿失禁是衰老的自然现象，而不是一种疾病吗？
① Agree / 同意
② Partially agree / 部分同意
③ Disagree / 不同意

Q8. Do you think little can be done about UI, so there is no need to spend time managing it?
您认为尿失禁没有太多干预手段，因此不需要花费时间管理吗？
① Agree / 同意
② Partially agree / 部分同意
③ Disagree / 不同意

Q9. Do you think patients should be guided to understand UI as a natural aging phenomenon and learn to adapt?
您认为应该引导患者将尿失禁视为自然衰老现象，并学会适应吗？
① Agree / 同意
② Partially agree / 部分同意
③ Disagree / 不同意

**Section 3: Clinical Practice Assessment (Scored on a 0-24 scale)**

**第3部分：临床实践评估（总分0-24）**

Q10. Do you confirm the presence of UI symptoms when consulting patients?
您在问诊时是否确认患者是否有尿失禁症状？
① Rarely / 很少
② Sometimes / 有时
③ Often / 经常

Q11. When patients mention UI, do you inquire about its impact on their quality of life?
当患者提及尿失禁时，您是否会询问其对生活质量的影响？
① Rarely / 很少
② Sometimes / 有时
③ Often / 经常

Q12. Do you provide questionnaires such as ICIQ-SF, I-QOL, or PISQ-12 to assess the impact of UI on various aspects of quality of life?
您是否提供 ICIQ-SF、I-QOL 或 PISQ-12 等问卷来评估尿失禁对生活质量的影响？
① Rarely / 很少
② Sometimes / 有时
③ Often / 经常

Q13. Are you confident in managing UI effectively?
您对有效管理尿失禁有信心吗？
① Agree / 同意
② Partially agree / 部分同意
③ Disagree / 不同意

Q14. Do you have a clear understanding of the causes of UI?
您对尿失禁的病因有清晰的认识吗？
① Agree / 同意
② Partially agree / 部分同意
③ Disagree / 不同意

Q15. Can you differentiate the type and severity of UI?
您能区分尿失禁的类型和严重程度吗？
① Agree / 同意
② Partially agree / 部分同意
③ Disagree / 不同意

Q16. Can you develop a diagnostic and treatment plan for UI patients?
您能为尿失禁患者制定诊断和治疗方案吗？
① Agree / 同意
② Partially agree / 部分同意
③ Disagree / 不同意

Q17. Do you perform specialized physical examinations (e.g., stress test) for UI patients?
您是否对尿失禁患者进行专门的体格检查（如压力试验）？
① Rarely / 很少
② Sometimes / 有时
③ Often / 经常

Q18. Do you understand how to conduct a pad test and explain it to patients?
您是否了解如何进行尿垫试验并向患者解释？
① Agree / 同意
② Partially agree / 部分同意
③ Disagree / 不同意

Q19. Do you understand how to conduct a voiding diary and explain it to patients?
您是否了解如何进行排尿日记并向患者解释？
① Agree / 同意
② Partially agree / 部分同意
③ Disagree / 不同意

Q20. Do you understand how to perform Kegel exercises and explain them to patients?
您是否了解如何进行凯格尔运动并向患者解释？
① Agree / 同意
② Partially agree / 部分同意
③ Disagree / 不同意

Q21. Do you have confidence in the effectiveness of health education for UI patients, such as teaching Kegel exercises and bladder training to improve symptoms?
您对尿失禁患者健康教育（如教授凯格尔运动和膀胱训练）改善症状的效果有信心吗？
① Agree / 同意
② Partially agree / 部分同意
③ Disagree / 不同意

**Additional Multiple-Choice Questions (Not Scored)**

**额外多选题（不计分）**

Q22. What are the reasons you do not perform specialized UI examinations?
您不进行尿失禁专门检查的原因是什么？

① Lack of proficiency in performing the procedure / 对该检查操作不熟练
② No perceived necessity for these examinations / 认为没有必要进行这些检查
③ Limited clinical space and conditions / 受限于临床场地和条件
④ Insufficient time during consultations / 问诊时间不足
⑤ Patients are elderly and have mobility difficulties / 患者年老、行动不便
⑥ Other reasons / 其他原因

Q23. What are the preferred assessment methods for UI?
您偏好的尿失禁评估方法有哪些？
① One-hour pad test / 1小时尿垫试验
② Urodynamic test / 尿流动力学检查
③ Urinalysis / 尿常规检查
④ Blood glucose test / 血糖检查
⑤ Urinary system ultrasound / 泌尿系统超声
⑥ Pelvic floor ultrasound / 盆底超声
⑦ Voiding diary / 排尿日记
⑧ Other / 其他

Q24. What factors contribute to poor efficacy to Kegel exercises and bladder training?
哪些因素导致凯格尔运动和膀胱训练疗效不佳？
① Insufficient consultation time to properly instruct patients / 问诊时间不足，难以详细指导
② Explanations are difficult for patients to understand / 讲解内容患者难以理解
③ Limited space and conditions for proper guidance / 场地和条件有限，难以规范指导
④ Lack of effective methods to supervise patients' adherence / 缺乏有效监督患者依从性的方法
⑤ Lack of tools to monitor treatment efficacy and adjust strategies accordingly / 缺乏监测疗效和调整策略的工具
⑥ Other reasons / 其他原因

Scoring System

Responses were scored using a three-point Likert scale.

For questions 1-12 and 17: Scores were assigned as 0, 1, and 2 for increasing levels of agreement or frequency.

For questions 13-16 and 18-21: Scores were reverse-coded as 2, 1, and 0.

The awareness and perceptions section had a maximum score of 18, the clinical practice section had a maximum score of 24, and the total questionnaire score ranged from 0 to 42. Higher scores reflect greater awareness of UI, more favorable attitudes toward UI management, and stronger adherence to recommended clinical practices.

评分系统

本问卷采用三点Likert评分法。

题目 Q1-Q12 和 Q17：分值为 0、1 和 2，得分越高代表认知度越高。

题目 Q13-Q16 和 Q18-Q21：采用反向评分，即 2、1 和 0。

认知与看法部分最高分 18 分，临床实践部分最高分 24 分，总问卷得分范围为 0-42。得分越高，表明对尿失禁的认知越深入、态度越积极、临床实践越符合推荐标准。
