# Supplementary material for: Urinary incontinence management in Chinese primary health institutions: findings from a regional survey
Source: PeerJ. 2026 Apr 7;14:e21079. doi: 10.7717/peerj.21079 (PMC13068013; doi:10.7717/peerj.21079)
Supplement: Supplemental Information 3 [file peerj-14-21079-s003.docx]

STROBE Statement—checklist of items that should be included in reports of observational studies

|  | Item No. | Recommendation | Page  No. | Relevant text from manuscript |
| --- | --- | --- | --- | --- |
| **Title and abstract** | 1 | (*a*) Indicate the study’s design with a commonly used term in the title or the abstract | 2 | “We conducted a cross-sectional survey...” |
|  |  | (*b*) Provide in the abstract an informative and balanced summary of what was done and what was found | 2-3 | Abstract – Background, Methods, Results, Conclusion |
| Introduction | | | |  |
| Background/rationale | 2 | Explain the scientific background and rationale for the investigation being reported | 3-4 | Introduction – Background/rationale section |
| Objectives | 3 | State specific objectives, including any prespecified hypotheses | 4 | Introduction – Last paragraph: this study aimed to evaluate the awareness, perceptions, and clinical practices related to UI among primary care providers in Fujian Province, China, and to identify associated factors and key barriers to effective management in both urban and rural settings. |
| Methods | | | |  |
| Study design | 4 | Present key elements of study design early in the paper | 5 | First paragraph of methods:A large-scale cross-sectional survey was conducted from March 2023 to September 2024 in county-level and lower-tier primary healthcare institutions across Fujian Province. |
| Setting | 5 | Describe the setting, locations, and relevant dates, including periods of recruitment, exposure, follow-up, and data collection | 5-6 | The above content was described in detail in the Study Design and Participants section and the Data Collection section of the Methods. |
| Participants | 6 | (*a*) *Cohort study*—Give the eligibility criteria, and the sources and methods of selection of participants. Describe methods of follow-up  *Case-control study*—Give the eligibility criteria, and the sources and methods of case ascertainment and control selection. Give the rationale for the choice of cases and controls  *Cross-sectional study*—Give the eligibility criteria, and the sources and methods of selection of participants | 5 | First paragraph of Design and Participants section: After explaining the study objectives and procedures, electronic invitations containing the anonymous survey link were distributed via professional WeChat groups organized by affiliated primary healthcare institutions. Participants were recruited using a convenience sampling strategy... |
|  |  | (*b*) *Cohort study*—For matched studies, give matching criteria and number of exposed and unexposed  *Case-control study*—For matched studies, give matching criteria and the number of controls per case |  |  |
| Variables | 7 | Clearly define all outcomes, exposures, predictors, potential confounders, and effect modifiers. Give diagnostic criteria, if applicable | 6 | The Data Collection section provides a detailed description of the questionnaire structure and the process of calculating the scores: “The questionnaire consisted of the following sections...” |
| Data sources/ measurement | 8* | For each variable of interest, give sources of data and details of methods of assessment (measurement). Describe comparability of assessment methods if there is more than one group | 6 | Data Collection section*–* Structured questionnaire |
| Bias | 9 | Describe any efforts to address potential sources of bias | 5 | The questionnaire was originally developed in Chinese based on the consensus of the Provincial Urogynecology Academic Committee, utilizing a two-round Delphi consultation process. |
| Study size | 10 | Explain how the study size was arrived at | 5 | Participants were recruited using a convenience sampling strategy among actively practicing gynecologists in these institutions. Although not probabilistic, this approach ensured broad coverage across all major counties in the province, thereby capturing a diverse and representative sample of primary care gynecologists from both urban and rural settings. |

Continued on next page

| Quantitative variables | 11 | Explain how quantitative variables were handled in the analyses. If applicable, describe which groupings were chosen and why | 6-7 | The Statistical Analysis section provides a detailed explanation of how the data were processed: “Continuous data was presented as mean ± standard deviation (SD) and between-group comparison was made using Student's t-test...” |
| --- | --- | --- | --- | --- |
| Statistical methods | 12 | (*a*) Describe all statistical methods, including those used to control for confounding | 6-7 | The Statistical Analysis section provides a detailed explanation of how the data were processed |
|  |  | (*b*) Describe any methods used to examine subgroups and interactions |  |  |
|  |  | (*c*) Explain how missing data were addressed |  |  |
|  |  | (*d*) *Cohort study*—If applicable, explain how loss to follow-up was addressed  *Case-control study*—If applicable, explain how matching of cases and controls was addressed  *Cross-sectional study*—If applicable, describe analytical methods taking account of sampling strategy |  |  |
|  |  | (*e*) Describe any sensitivity analyses |  |  |
| Results | | | | |
| Participants | 13* | (a) Report numbers of individuals at each stage of study—eg numbers potentially eligible, examined for eligibility, confirmed eligible, included in the study, completing follow-up, and analysed | 7 | First paragraph of Results:“A total of 1,427 primary care gynecologists participated, with 75.5% practicing in urban areas. The mean age was 36.61 ± 9.29 years, and 81.7% were female...” |
|  |  | (b) Give reasons for non-participation at each stage | Not available |  |
|  |  | (c) Consider use of a flow diagram | Not available |  |
| Descriptive data | 14* | (a) Give characteristics of study participants (eg demographic, clinical, social) and information on exposures and potential confounders | 7 | Table 1 provides a detailed results |
|  |  | (b) Indicate number of participants with missing data for each variable of interest | Not available |  |
|  |  | (c) *Cohort study*—Summarise follow-up time (eg, average and total amount) | Not available |  |
| Outcome data | 15* | *Cohort study*—Report numbers of outcome events or summary measures over time | Not available |  |
|  |  | *Case-control study—*Report numbers in each exposure category, or summary measures of exposure | Not available |  |
|  |  | *Cross-sectional study—*Report numbers of outcome events or summary measures | *7* | Table 2-4 provides a detailed results |
| Main results | 16 | (*a*) Give unadjusted estimates and, if applicable, confounder-adjusted estimates and their precision (eg, 95% confidence interval). Make clear which confounders were adjusted for and why they were included | 9 | Table 3 “Regression analysis revealed that higher awareness scores were significantly associated with female gender (P = 0.038), bachelor's (P = 0.007) and master's degrees (P = 0.006). Higher clinical practice scores correlated positively with age (P = 0.011) and female gender (P < 0.001)” |
|  |  | (*b*) Report category boundaries when continuous variables were categorized |  |  |
|  |  | (*c*) If relevant, consider translating estimates of relative risk into absolute risk for a meaningful time period |  |  |

Continued on next page

| Other analyses | 17 | Report other analyses done—eg analyses of subgroups and interactions, and sensitivity analyses | 8 | Questionnaire Reliability and Validity: “Overall Cronbach's α indicated high reliability (overall Cronbach's α = 0.84).....” |
| --- | --- | --- | --- | --- |
| Discussion | | | | |
| Key results | 18 | Summarise key results with reference to study objectives | 10 | First paragraph of discussion:“This study systematically evaluated the awareness, perceptions, and clinical practices of primary care gynecologists” |
| Limitations | 19 | Discuss limitations of the study, taking into account sources of potential bias or imprecision. Discuss both direction and magnitude of any potential bias | 13 | “This study has several limitations. First, self-reported survey data may be subject to response bias, as physicians who are more knowledgeable or confident in UI management may have been more likely to participate, potentially leading to an overestimation of overall competence. ” |
| Interpretation | 20 | Give a cautious overall interpretation of results considering objectives, limitations, multiplicity of analyses, results from similar studies, and other relevant evidence | 10-13 | The above content was discussed in detail in the Discussion section of the manuscript. |
| Generalisability | 21 | Discuss the generalisability (external validity) of the study results | 13 | The issue of adaptability requiring further confirmation was mentioned in the Limitations section of the discussion: “Third, while the questionnaire was developed based on expert opinion, it has not undergone formal psychometric validation. Future studies should aim to develop and validate standardized tools tailored to the Chinese primary care context. ” |
| Other information | |  | | |
| Funding | 22 | Give the source of funding and the role of the funders for the present study and, if applicable, for the original study on which the present article is based | Not available |  |

*Give information separately for cases and controls in case-control studies and, if applicable, for exposed and unexposed groups in cohort and cross-sectional studies.

**Note:** An Explanation and Elaboration article discusses each checklist item and gives methodological background and published examples of transparent reporting. The STROBE checklist is best used in conjunction with this article (freely available on the Web sites of PLoS Medicine at http://www.plosmedicine.org/, Annals of Internal Medicine at http://www.annals.org/, and Epidemiology at http://www.epidem.com/). Information on the STROBE Initiative is available at www.strobe-statement.org.
